# Association of *S100B* polymorphisms and serum S100B with risk of ischemic stroke in a Chinese population

**DOI:** 10.1038/s41598-018-19156-w

**Published:** 2018-01-17

**Authors:** Yu-Lan Lu, Rong Wang, Hua-Tuo Huang, Hai-Mei Qin, Chun-Hong Liu, Yang Xiang, Chun-Fang Wang, Hong-Cheng Luo, Jun-Li Wang, Yan Lan, Ye-Sheng Wei

**Affiliations:** 1 Department of Clinical Laboratory, Affiliated Hospital of Youjiang Medical University for Nationalities, No.18 Zhongshan Road II, Baise, 533000 Guangxi China; 2 Department of Dermatology, Affiliated Hospital of Youjiang Medical University for Nationalities, No.18 Zhongshan Road II, Baise, 533000 Guangxi China

## Abstract

The levels of serum S100B were elevated in patients with ischemic stroke (IS), which may be a novel biomarker for diagnosing IS. The aim of this study was to investigate the association of *S100B* polymorphisms and serum S100B with IS risk. We genotyped the *S100B* polymorphisms rs9722, rs9984765, rs2839356, rs1051169 and rs2186358 in 396 IS patients and 398 controls using polymerase chain reaction-single base extension (SBE-PCR). Serum S100B levels were measured by enzyme-linked immunosorbent assay (ELISA). Rs9722 was associated with an increased risk of IS (AA vs. GG: adjusted OR = 2.172, 95% CI, 1.175–4.014, *P* = 0.013; dominant: adjusted OR = 1.507, 95% CI, 1.071–2.123, *P* = 0.019; recessive: adjusted OR = 1.846, 95% CI, 1.025–3.323, *P* = 0.041; additive: adjusted OR=1.371, 95% CI, 1.109-1.694, P = 0.003). The A-C-C-C-A haplotype was associated with an increased risk of IS (OR = 1.325, 95% CI, 1.035–1.696, *P* = 0.025). In addition, individuals carrying the rs9722 GA/AA genotypes had a higher serum S100B compared with the rs9722 GG genotype in IS patients (*P* = 0.018). Our results suggest that the *S100B* gene rs9722 polymorphism may contribute to the susceptibility of IS, probably by promoting the expression of serum S100B.

## Introduction

Stroke is a multi-factorial disease that constitutes one of the leading causes of adult disability worldwide^1–3^. In recent years, the incidence of stroke has increased dramatically in China^4^. Approximately 80% of strokes are ischemic in origin. Ischemic stroke (IS) is the result of interrupted blood flow within the area of an occluded blood vessel, causing local brain tissue to become deprived of oxygen, ending in malacia and necrosis. Several risk factors have been identified to contribute to the pathogenesis of IS, including age, gender, obesity, hypertension, diabetes, smoking and dyslipidaemia^5^. However, these conventional risk factors do not fully account for the overall risk of IS. Several lines of evidence have indicated that genetic factors are also involved in the development of IS^6,7^. To date, the possible relationship of IS with altered transcription of genes has not been ruled out.

S100 calcium-binding protein B (S100B) belongs to the large superfamily of S100, which is mainly expressed by astrocytes in the brain and plays a crucial role in cell proliferation, differentiation, apoptosis, signal transduction, cellular energy and metabolism^8,9^. Furthermore, by interacting with the receptor for advanced glycation end products (RAGE), S100B can activate microglial cells and stimulate the secretion of inflammatory cytokines, such as tumour necrosis factor-α (TNF-α), interleukin-1β (IL-1β) and the chemokine 22 (CCL22), and upregulate the expression of the proinflammatory enzyme COX-2^10,11^. These cytokines have been previously reported to play a role in the pathogenesis of IS^12–16^. More recently, increasing evidence has identified that serum S100B levels may be used as a potential biomarker for cardiovascular diseases^17–22^. In addition, evidence from clinical studies and animal models have suggested that elevated levels of serum S100B play a vital role in the development of IS^23–26^. Taken together, these findings indicate that S100B may represent a promising candidate for the treatment of IS.

Single nucleotide polymorphisms (SNPs) are the most common variants in human genomes and have been used frequently as genetic markers in genome-wide association studies (GWAS)^27^. The human *S100B* gene is located on chromosome 21q22.3, which consists of 3 exons and 2 introns. Previously, a number of studies have indicated that *S100B* polymorphisms may modulate an individual’s susceptibility to several human diseases, such as schizophrenia, dyslexia, autism and bipolar affective disorder^28–31^. However, to our knowledge, no study has investigated the associations between *S100B* polymorphisms and IS susceptibility. Therefore, the aim of this study was to investigate the association of the five SNPs in the *S100B* gene with susceptibility to IS in a Chinese population. Moreover, the effect of *S100B* polymorphisms on the levels of serum S100B was also assessed.

## Results

### Clinical characteristics of the study participants

The clinical characteristics of IS patients and controls are summarized in Table 1. There were no significant differences between the two groups based on age, gender and TCH (*P* > 0.05). The frequencies of hypertension, diabetes mellitus and smoker in IS patients were significantly higher than those in controls (*P* < 0.05). Increased levels of TG, LDL-C, VLDL-C and lower levels of HDL-C were observed in IS patients compared with controls (*P* < 0.001).

**Table 1:** Clinical characteristics of the study population.

Variables	Controls (n = 398)	IS patients (n = 396)	*P* value
Age, years (mean ± SD)	58.09 ± 8.76	59.16 ± 9.18	0.093
Gender (M/F)	231/167	252/144	0.106
Hypertension, n (%)	88 (22.1%)	161 (40.6%)	<0.001
Diabetes mellitus, n (%)	41 (10.3%)	64 (16.2%)	0.015
Smoker, n (%)	62 (15.6%)	116 (29.3%)	<0.001
TCH, mmol/L	4.84 ± 0.74	4.78 ± 1.23	0.390
TG, mmol/L	1.24 ± 0.74	1.60 ± 1.09	<0.001
HDL-C, mmol/L	1.66 ± 0.39	1.26 ± 0.35	<0.001
LDL-C, mmol/L	2.47 ± 0.56	2.64 ± 0.89	<0.001
VLDL-C, mmol/L	0.57 ± 0.36	0.73 ± 0.49	<0.001

IS, ischemic stroke; SD, standard deviation; M, male; F, female; TCH, total cholesterol; TG, triglyceride; HDL-C, high density lipoprotein-cholesterol; LDL-C, low density lipoprotein-cholesterol; VLDL-C, very low density lipoprotein-cholesterol.

### Association of *S100B* polymorphisms with IS risk

All five SNP genotypes were in HWE among control subjects (*P* > 0.05). The association between *S100B* polymorphisms and risk of IS under genotype and genetics models analysis are shown in Table 2. We observed that the rs9722 AA genotype was associated with an increased risk of IS compared with the GG genotype, even after adjusting for age, gender, hypertension, diabetes mellitus, smoker, TCH, TG, HDL-C, LDL-C and VLDL-C (AA vs. GG: adjusted OR = 2.172, 95% CI, 1.175–4.014, *P* = 0.013). Similarly, a significantly increased risk was also observed in the dominant model (GA/AA vs. GG: adjusted OR = 1.507, 95% CI, 1.071–2.123, *P* = 0.019), recessive model (AA vs. GA/GG: adjusted OR = 1.846, 95% CI, 1.025–3.323, *P* = 0.041) and additive model (A vs. G: adjusted OR=1.371, 95% CI, 1.109-1.694, P = 0.003). However, after correction for multiple comparisons, all associations described above lost statistical significance. Studies with greater sample sizes are needed to confirm these associations.

**Table 2:** Association between the *S100B* polymorphisms and risk of IS.

SNPs	Controls (n = 398)	IS patients (n = 396)	AOR (95% CI)^†^	*P* ^†^	*P*_*BH*_
**rs9722**					
GG	197 (49.5)	163 (41.2)	1.000 (ref)		
GA	175 (44.0)	186 (47.0)	1.392 (0.973–1.993)	0.071	0.213
AA	26 (6.5)	47 (11.9)	2.172 (1.175–4.014)	0.013	0.095
Dominant			1.507 (1.071–2.123)	0.019	0.095
Recessive			1.846 (1.025–3.323)	0.041	0.154
Additive			1.371 (1.109–1.694)	0.003	0.045
**rs9984765**					
TT	190 (47.7)	176 (44.4)	1.000 (ref)		
CT	177 (44.5)	179 (45.2)	1.290 (0.902–1.845)	0.163	0.348
CC	31 (7.8)	41 (10.4)	1.497 (0.817–2.743)	0.192	0.348
Dominant			1.325 (0.941–1.864)	0.107	0.268
Recessive			1.320 (0.740–2.354)	0.347	0.377
Additive			1.146 (0.927–1.416)	0.209	0.348
**rs2839356**					
TT	196 (49.2)	182 (46.0)	1.000 (ref)		
CT	174 (43.7)	178 (44.9)	1.194 (0.837–1.703)	0.327	0.377
CC	28 (7.1)	36 (9.1)	1.080 (0.574–2.032)	0.812	0.508
Dominant			1.175 (0.837–1.650)	0.352	0.377
Recessive			0.992 (0.539–1.824)	0.979	0.587
Additive			1.115 (0.900–1.382)	0.320	0.377
**rs1051169**					
GG	164 (41.2)	153 (38.6)	1.000 (ref)		
CG	178 (44.7)	186 (47.0)	1.149 (0.795–1.660)	0.460	0.406
CC	56 (14.1)	57 (14.4)	1.152 (0.684–1.942)	0.595	0.406
Dominant			1.150 (0.812–1.627)	0.432	0.406
Recessive			1.069 (0.660–1.731)	0.787	0.508
Additive			1.064 (0.868–1.304)	0.551	0.406
**rs2186358**					
AA	328 (82.4)	336 (84.8)	1.000 (ref)		
AC	63 (15.8)	56 (14.2)	0.876 (0.549–1.398)	0.579	0.406
CC	7 (1.8)	4 (1.0)	0.608 (0.130–2.857)	0.529	0.406
Dominant			0.853 (0.543–1.340)	0.490	0.406
Recessive			0.621 (0.132–2.910)	0.545	0.406
Additive			0.821 (0.580–1.162)	0.265	0.377

IS, ischemic stroke; OR, odds ratio; 95% CI, 95% confidence interval. ^†^Adjusted by age, gender, hypertension, diabetes mellitus, smoker, TCH, TG, HDL-C, LDL-C and VLDL-C. P_BH_: *P* values corrected by Benjamin-Hochberg (B-H) method.

### Distribution of the *S100B* gene rs9722 polymorphism in different populations

Because rs9722 may play an important role in the development of IS, we then performed a comparison of the genotype distribution of rs9722 in different populations (Table 3**)**. The results showed that the genotype distribution of rs9722 in our study was significantly different compared with HM-HCB, HM-JPT, HM-CEU, HM-YRI, HM-ASW, HM-LWK, HM-MEX, HM-MKK and HM-TSI (*P* < 0.05). However, no significant difference was found when comparing with HM-CHB, HM-CHD and HM-GIH (*P* > 0.05).

**Table 3:** Distribution of the rs9722 polymorphism in different populations.

Group	N	Genotype (%)			Allele (%)		Ethnicity
		GG	GA	AA	G	A	
Our data	398	197 (49.5)	175 (44.0)	26 (6.5)	569 (71.5)	227 (28.5)	Guangxi
HM-CHB	82	46 (56.1)	26 (31.7)	10 (12.2)	118 (72.0)	46 (28.0)	Asian
HM-HCB^*^	84	26 (30.9)	44 (52.4)	14 (16.7)	96 (57.1)	72 (42.9)	Asian
HM-CHD	170	82 (48.2)	78 (45.9)	10 (5.9)	242 (71.2)	98 (28.8)	Asian
HM-JPT^*^	172	60 (34.9)	98 (57.0)	14 (8.1)	218 (63.4)	126 (36.6)	Asian
HM-CEU^*^	226	184 (81.4)	40 (17.7)	2 (0.9)	408 (90.3)	44 (9.7)	European
HM-YRI^*^	226	46 (20.3)	124 (54.9)	56 (24.8)	216 (47.8)	236 (52.2)	African
HM-ASW^*^	98	36 (36.7)	50 (51.0)	12 (12.3)	122 (62.2)	74 (37.8)	African
HM-GIH	174	90 (51.7)	72 (41.4)	12 (6.9)	252 (72.4)	96 (27.6)	Asian
HM-LWK^*^	176	64 (36.4)	82 (46.6)	30 (17.0)	210 (59.7)	142 (40.3)	Asian
HM-MEX^*^	100	78 (78.0)	22 (22.0)	—	178 (89.0)	22 (11.0)	America
HM-MKK^*^	284	78 (27.5)	144 (50.7)	62 (21.8)	300 (52.8)	268 (47.2)	African
HM-TSI^*^	176	154 (87.5)	20 (11.4)	2 (1.1)	328 (93.2)	24 (6.8)	European

^*^P < 0.05 comparing with our present data; HM: Haplotype Map; CHB: Han Chinese in Beijing, China; HCB: Han Chinese in Beijing, China; CHD: Chinese in Metropolitan Denver, Colorado; JPT: Japanese in Tokyo, Japan; CEU: Utah residents with northern and western European ancestry; YRI: Yoruba in Ibadan, Nigeria; ASW: African ancestry in Southwest USA; GIH: Gujarati Indians in Houston, Texas; LWK: Luhya in Webuye, Kenya; MEX: Mexican ancestry in Los Angeles, California; MKK: Maasai in Kinyawa, Kenya; TSI: Toscans in Italy.

### Haplotype analysis of the *S100B* gene

Haplotype analysis was performed and the possible five haplotype frequencies are shown in Table 4. The results showed that rs9984765 was in strong linkage disequilibrium (LD) with rs2839356 (D′ = 0.931) and rs1051169 (D′ = 0.882). Similarly, rs2839356 was in strong LD with rs1051169 (D′ = 0.852) and rs2186358 (D′ = 0.805). Moreover, we found that the A-C-C-C-A haplotype was associated with an increased risk of IS (OR = 1.325, 95% CI, 1.035–1.696, *P* = 0.025). The G-T-T-C-C haplotype was associated with a decreased risk of IS (OR = 0.480, 95% CI, 0.275–0.838, *P* = 0.008).

**Table 4:** Haplotype analysis of the *S100B* polymorphisms with risk of IS.

Haplotype	Controls (2n = 796)	IS patients (2n = 792)	OR (95% CI)	*P* value
G T T G A	414 (52.0)	385 (48.6)	0.899 (0.726–1.113)	0.329
A C C C A	152 (19.1)	183 (23.1)	1.325 (1.035–1.696)	0.025
G C C C A	47 (5.9)	41 (5.2)	0.891 (0.579–1.372)	0.600
A T T G A	46 (5.7)	51 (6.5)	1.163 (0.769–1.760)	0.474
G T T C C	40 (5.0)	19 (2.4)	0.480 (0.275–0.838)	0.008

IS, ischemic stroke; OR, odds ratio; 95% CI, 95% confidence interval.

### Association of *S100B* polymorphism and serum S100B levels

**Figure 1:**
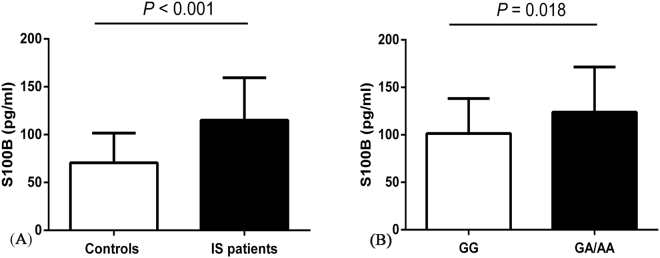
ELISA detection of serum S100B levels. **(A)** There were significant differences in the levels of serum S100B between IS patients [(115.03 ± 44.42) pg/mL; n = 44] and controls [(70.53 ± 30.98) pg/mL; n = 44], (P < 0.001). **(B)** Increased levels of serum S100B in IS patients carrying the rs9722 GA/AA genotypes [(123.98 ± 47.42) pg/mL; n = 52] compared with those carrying the rs9722 GG genotype [(101.33 ± 36.98) pg/mL; n = 36)], (*P* = 0.018). Data are presented as the mean ± standard error.

We then investigated the association between *S100B* polymorphisms and serum S100B levels. As shown in Fig. 1, the levels of serum S100B were significantly up- regulated in IS patients compared with controls [(115.03 ± 44.42) pg/mL vs. (70.53 ± 30.98) pg/mL, *P* < 0.001]. Notably, we found that patients carrying the rs9722 GA/AA genotypes had a higher expression of serum S100B compared with those carrying the rs9722 GG genotype [(123.98 ± 47.42) pg/mL vs. (101.33 ± 36.98) pg/mL, *P* = 0.018].

## Discussion

To our knowledge, this is the first report to determine whether *S100B* polymorphisms and the levels of serum S100B are associated with IS in the Chinese population. In this study, we observed that the rs9722 AA genotype, dominant model, recessive model and additive model were associated with significantly increased risk of IS. An increased risk was also observed in the haplotype analysis. Moreover, we found that the levels of serum S100B were significantly up-regulated in IS patients compared with controls. Interestingly, the rs9722 GA/AA genotypes corresponded to higher levels of serum S100B. The statistical power of the study was calculated to be 91% to detect the association between rs9722 polymorphism and IS risk in a sample size of 794 participants (396 IS patients and 398 controls), assuming an OR of 1.6 and α of 0.05 (PASS 15.0 software). Therefore, these findings indicate that the *S100B* gene rs9722 polymorphism may serve as a novel genetic marker of susceptibility to IS in the Chinese population.

Stroke is one of the major causes of death and long-term disability worldwide. Globally, there are >50 million stroke patients, producing an immense burden on the economic and healthcare infrastructure^32^. To date, however, the exact aetiology and pathogenetic mechanisms of IS remain unclear. Recently, increasing evidence has indicated that serum S100B levels may be used as a novel biomarker for IS^33–35^. Nevertheless, the mechanisms leading to elevated serum S100B are unknown, but this is believed to lead to aggravation of the development of IS. In the present study, our results also showed that the levels of serum S100B in IS patients were significantly higher than in controls. The results of our study suggest that S100B may play a crucial role in the aetiology of IS.

Recently, several studies have been conducted to investigate the effect of the rs9722 polymorphism on human diseases. Matsson *et al*.^29^ reported that the rs9722 polymorphism was associated with dyslexia, and the T allele was suggested as a risk factor for the development of dyslexia. Hohoff *et al*.^36^ found that the rs9722 AA genotype was significantly associated with the high expression of S100B in the prefrontal cortex or peripheral blood. Li *et al*.^37^ demonstrated that the rs9722 T allele was associated with the risk of severe hand, foot and mouth disease. Similarly, a case-control study conducted by Liu *et al*.^38^ reported that the rs1051169-rs9722 (G-C) haplotype may have a possible susceptibility to increase the expression of serum S100B. In contrast, Yang *et al*.^39^ showed that the rs9722 polymorphism was not correlated with the risk of major depressive disorder in a Chinese population. To date, no association study has been reported on the association between the rs9722 polymorphism and IS. However, in this study, we found that the rs9722 AA genotype, dominant, recessive and additive model display an increased risk of IS. Additionally, the levels of serum S100B were found to be elevated in IS patients. Interestingly, we observed that individuals carrying the rs9722 GA/AA genotypes had a higher serum S100B compared with the rs9722 GG genotype in IS patients. In summary, these results suggest that the *S100B* gene rs9722 polymorphism may be responsible for susceptibility to IS, probably through up-regulation of the expression of serum S100B.

Until now, very limited data have been reported on the association of rs9984765, rs2839356 and rs2186358 polymorphisms with disease susceptibility. A previous study conducted by Hohoff *et al*.^36^ reported that the T-G-G-A (rs2186358-rs11542311-rs2300403-rs9722) haplotype was associated with elevated levels of serum S100B. Meanwhile, the G-A-T-C (rs11542311-rs2839356-rs9984765-rs881827) haplotype was associated with increased expression of *S100B* mRNA in postmortem frontal cortices. Regarding the rs1051169 polymorphism, Guo *et al*.^40^ have tried to detect the association of the rs1051169 polymorphism with Parkinson’s disease in a Chinese population, but failed to have a positive result. However, Liu *et al*.^38^ found that the rs1051169 polymorphism was associated with an increased risk of schizophrenia in the Chinese population.

In the present study, we failed to find any association of the rs9984765, rs2839356, rs1051169 and rs2186358 polymorphisms with IS risk. Two possibilities should be taken into account to explain the negative results. First, it may be because of genetic trait differences, as we know that genetic polymorphisms in human genes are distinct in specific populations, various ethnicities and geographic regions. Data from Table 5 support this viewpoint, we observed that the genotype distribution of rs9722 in our study showed significant differences compared with the HM-HCB, HM-JPT, HM-CEU, HM-YRI, HM-ASW, HM-LWK, HM-MEX, HM-MKK and HM-TSI populations, but was similar to the HM-CHB, HM-CHD and HM-GIH populations. Secondly, stroke is a multi-factorial disease that is regulated by genetic and environmental factors; thus, individual exposure to different environmental factors and genetic susceptibility might have caused different results.

**Table 5:** The primer sequences used for detecting five SNPs of the *S100B* gene.

SNP ID	Position	PCR primers
rs9722	48019239	F: 5′-ACAACACGGCTGGAAAGCTCAG-3′
		R: 5′-GATGGAGACGGCGAATGTGACT-3′
		EF: 5′-TTTTTTTTTTTTTTTTTTGCCAAACCTTTCCTGTAACAGAGA-3′
rs9984765	48021655	F: 5′-CTCCACAGAGCCTCCTGCAAAG-3′
		R: 5′-CCTGGCACATGGATGAATGACC-3′
		EF: 5′-TTTTTTTTTTTTACCATGACTGGCTTAACTGAGG-3′
rs2839356	48022144	F: 5′-TCACCTTCAGGGCAGCTGAGAA-3′
		R: 5′-TGGAAGGGAGGGAGACAAGCAC-3′
		EF: 5′-TTTTTTTTTTTTTTTTTTTTTTTTAAGATAAAGCCAGTGTACAGATGGAT-3′
rs1051169	4802230	F: 5′-TCACCTTCAGGGCAGCTGAGAA-3′
		R: 5′-TGGAAGGGAGGGAGACAAGCAC-3′
		EF: 5′-TTTTTTTTTTTTTTTTTTTTTTTTTTTTTTTTTTTTTTTCACAAGCTGAAGAAATCCGAACT-3′
rs2186358	48022375	F: 5′-GACATCCTAGGGGCTCGCAAAG-3′
		R: 5′-CCCCTTGTCTGGGTTGAGGTCT-3′
		EF: 5′-TTTTTTTTTTTTTTTTTTTTTTTTTTTTTTTAGTTGCCTTCTCATCTATACCTCATC-3′

F: forward primer; R: reverse primer; EF: Extended primer.

S100B, produced mainly by activated astrocytes, has already been confirmed to participate in regulating cell proliferation, differentiation and apoptosis^41^. Previous studies have indicated that extracellular S100B binds to its membrane receptor RAGE and then activates a series of cellular signalling pathways and leads to the production of TNF-α, IL-1β, IL-6 and VCAM-1^11,42,43^. Serum levels of IL-1β and IL-6 were significantly increased in IS patients, with a function of promoting IS progression^13^. TNF-α has been previously reported to play a key role in the pathogenesis of IS^44,45^. Cao *et al*.^46^ reported that S100B can promote vascular smooth muscle cell (VSMC) proliferation, causing neointimal formation, whereas secreted S100B from VSMCs may block re-endothelialisation and impair vascular repair. Beer *et al*.^47^ reported that serum-S100B was positively correlated with plasma high-sensitivity C-reactive protein. In addition, clinical and experimental studies have demonstrated that the levels of serum S100B were elevated in patients with IS^33,34,48^. Furthermore, a previous study found that knockdown of S100B in atherosclerotic mice can improve the brain’s recovery function and reduced infarctions^49^. Given the crucial role of S100B in IS pathogenesis, the positive results of our present study were biologically reasonable.

There are several limitations in our study. First, the relatively small sample size may limit the statistical power of our study. Further large-scale studies still need to be performed. Second, because this is a hospital-based case-control study, we cannot rule out the possibility of selection bias. Finally, our study subjects are all Chinese; thus, the results cannot be directly applicable to other ethnic groups.

In summary, our study provides evidence that the polymorphism of rs9722 in the *S100B* gene is associated with IS in the Chinese population. In the future, further studies with a larger sample size in diverse ethnic groups should be performed to confirm these findings.

## Materials and Methods

### Study population

The procedure was approved by the Review Boards of Affiliated Hospital of Youjiang Medical College for Nationalities. The study was performed in accordance with the relevant guidelines. All participators have written informed consent before participating in this study. The study subjects included 396 patients with IS and 398 controls. All IS patients were collected from the Department of Neurology, Affiliated Hospital of Youjiang Medical University for Nationalities, Guangxi, China between January 2013 and September 2016. IS was defined as a focal or global neurological deficit of sudden onset lasting more than 24 h caused by cerebral ischemia. All IS patients were diagnosed according to clinical symptoms, physical examination and cranial computed tomography (CT) or magnetic resonance imaging (MRI). Patients with haemorrhagic stroke, traumatic brain injuries, cardiogenic thrombosis and tumours were excluded in our study. The control subjects were selected from the Health Medical Center of the hospital during the same period. Individuals with tumours, autoimmune diseases, genetic disease, liver ailments and haematological diseases were excluded in this study. Clinical information, such as hypertension, diabetes, smoker, fasting serum levels of total cholesterol (TCH), triglyceride (TG), high density lipoprotein-cholesterol (HDL-C), low density lipoprotein-cholesterol (LDL-C) and very low density lipoprotein-cholesterol (VLDL-C), was abstracted from the medical record review of our hospital. The controls were frequency matched to cases in terms of age and gender. All study subjects were unrelated Han Chinese.

### DNA Extraction and Genotyping

Blood samples from all subjects were collected in EDTA-containing tubes. Genomic DNA was isolated from peripheral blood mononuclear cells using a DNA extraction kit (QIANGEN, China) according to the manufacturer’s instructions and then stored at −70 °C for later use. Primer probes were designed using Primer Express Software (version 3.0) and synthesized and supplied by Applied Biosystems (United States). Primer sequences are presented in Table 5. Genotyping was performed using SBE-PCR. The PCRs were performed in a total volume of 20 μL containing 3.0 mmol/L Mg^2+^, 0.3 mmol/L dNTP, 1 U HotStarTaq polymerase (QIANGEN, China), 1 μL genomic DNA, 1 μL PCR primer and 1× GC-I buffer (Takara). The PCR conditions included an initial denaturation step at 94 °C for 20 s, followed by 35 cycles with 20 s of denaturation at 94 °C, 30 s of annealing at 59 °C and 1.5 min of elongation at 72 °C, followed by a final elongation step of 72 °C for 2 min. PCR products were digested with Shrimp enzyme (SAP, from Promega) and excision enzyme (EXO I, from Epicentre). An ABI PRISM 3730XL analyser (PE Applied Biosystems, Foster City, CA, USA) sequenced the PCR products. The samples were reanalysed and verified by DNA sequencing if conflict results occurred. In addition, approximately 10% of all samples were randomly selected to be confirmed by DNA sequencing, and the results were 100% consistent.

### Serum S100B determination

Serum samples from IS patients and control subjects were separated from peripheral venous blood at room temperature and stored at −70 °C until use. The quantity determination of the levels of serum S100B was performed by ELISA kits (Human S100B, BioVendor, No: RD192090100R) following the manufacturer’s protocol. The developed colour reaction was measured as OD450 units on an ELISA reader (RT-6000, China). The concentration of serum S100B was determined using a standard curve constructed with the kit’s standards over the range of 10–320 pg/mL.

### Statistical analysis

All data were analysed with the software Statistical Package for Social Science (SPSS) for Windows, version 17.0 (SPSS, Inc., Chicago, USA). Hardy-Weinberg equilibrium (HWE) was tested by the chi-square test. Categorical variables were expressed as proportions and compared using the chi-squared test. Continuous variables were displayed the as mean ± SD. If the data were normally distributed, Student’s t-test was used; otherwise, the Mann-Whitney U test was used. The odds ratio (OR) and 95% confidence intervals (CI) were calculated to provide a measure of the strength of the *S100B* polymorphisms on IS risk. Logistic regression analysis was performed to estimate the putative association between the SNPs and the risk of IS while adjusting for age, sex, hypertension, diabetes, smoker, TCH, TG, HDL-L, LDL-L and VLDL-L. We carried out multiple hypothesis testing using the Benjamini-Hochberg method to control the false discovery rate (FDR) in the unconditional logistic regression analysis. Haplotype analysis was performed on an online tool SHEsis^50^. Statistical significance was set at *P* < 0.05.
